# Health state utilities of a population of Nigerian hypertensive patients

**DOI:** 10.1186/1756-0500-4-528

**Published:** 2011-12-12

**Authors:** Obinna I Ekwunife, Cletus N Aguwa, Maxwell O Adibe, Ebenezer Barikpaoar, Chioma J Onwuka

**Affiliations:** 1 Department of Clinical Pharmacy and Pharmacy Management, Faculty of Pharmaceutical Sciences, University of Nigeria Nsukka, Nsukka, Enugu, Nigeria; 2 Department of Clinical Pharmacy, Faculty of Pharmaceutical Sciences, University of Port Harcourt, Port Harcourt, Rivers, Nigeria; 3 Pharmaceutical Outcomes Research Team, Department of Clinical Pharmacy and Pharmacy Management, Faculty of Pharmaceutical Sciences, University of Nigeria Nsukka, Nsukka, Enugu, Nigeria; 4 The School of Pharmacy, University of London, 29/39 Brunswick Square, London WC1N 1AX, UK

**Keywords:** Quality of life, Hypertension, Cardiovascular disease (CVD), Validation, Nigeria

## Abstract

**Background:**

Establishment of the health impact of hypertension on quality of life of Nigerians is a step towards controlling the disease. The study aimed to provide a Nigerian specific reference list of utility scores of hypertensive patients with various interacting conditions.

**Findings:**

An interviewer-based, cross-sectional study was conducted using hypertensive patients in two purposively selected tertiary hospitals located in South-Eastern Nigeria. Health Utility Index Mark 3 (HUI3) was used.

A total of 384 participants with either hypertension alone or with hypertension-associated complications were interviewed in the two tertiary hospitals.

The overall mean utility score was 0.35 +/- 0.42. Patients with hypertension alone had the highest overall mean utility score (0.57 +/- 0.29) while hypertensive patients with stroke had the lowest overall mean score (0.04 +/- 0.36). Being a male, increase in age and mean arterial blood pressure, emergency visit and loss of work due to illness were associated with significant decrease in overall utility scores.

**Conclusions:**

This study presented a reference for health state utilities of a population of Nigerian hypertensive patients.

## Findings

Hypertension is a major cardiovascular risk factor. Direct correlations between blood pressure (BP) values and risk of cardiovascular diseases (CVDs) have been established based principally on epidemiologic data. Beginning at a benchmark BP of 115/75 mmHg, the risk of CVD doubles with every increment of 20/10 mmHg [1]. This finding is not peculiar to whites in developed nations. The INTERHEART study showed that hypertension is a strong contributor to the hazards of CVD in black Africans, with odds ratio of 7.0 versus 2.3 in other ethnic groups [2].

There is growing evidence that prevalence of hypertension is on the increase in most sub-Saharan African countries including Nigeria [3]. The current prevalence in many developing countries, particularly in urban societies, is said to be already as high as those seen in developed countries [4,5]. The growing trend in prevalence of hypertension in Africa is supported by a number of reviews. A systematic review published in 2007, showed that prevalence of hypertension in these regions of the world could range from 14.2% to 39.1% (all age standardized prevalence rates) [6]. Nigeria is not left out in this epidemiological shift as prevalence of hypertension appears to be increasing in the nation [7].

Establishing the health impact of hypertension on quality of life of Nigerians is a step towards checking the disease. This will highlight the need to address the emerging burden. For this, utility measures are often employed. Economic evaluation of programmes to detect and treat hypertension by improving awareness, treatment and adherence and level of control has been identified as a research priority in hypertension for developing nations such as Nigeria [3] and utility estimates are necessary for such evaluations. Utility estimates which are preference based are employed as quality weights for calculating the number of quality-adjusted life-years (QALYs) gained in cost-utility analyses.

The aim of this study is to provide a Nigerian specific reference list of utility scores of hypertensive patients with various interacting cardiovascular conditions. A catalogue of utilities scores of Nigerian hypertensive patients would enable more efficient economic analyses of health interventions.

## Methods

A descriptive study was conducted to assess a cross-section of hypertensive patients with or without hypertension-associated complications [8] in two purposively selected tertiary hospitals located in South-Eastern Nigeria. These hospitals were University of Nigeria Teaching Hospital Ituku-Ozalla and Ebonyi State University Teaching Hospital Abakaliki. The study was conducted from August 2010 to January 2011. Study participants included all the patients attending the outpatient cardiology clinics in the two hospitals during the period of study. Participants were included in the study if they were diagnosed with hypertension (≥ 140/90 mmHg) and/or hypertension-associated complications as specified in their medical record, consented to participate, and were sufficiently literate in English to answer questions. Subjects were excluded if they had more than one cormobidity, were at the end stage of sickness (as indicated in their medical record), declined enrollment or were unable or unwilling to give informed consent. The interviewer-based, exit study was administered by 3 final year pharmacy students after having been trained on how to administer the questionnaire.

The HUI3 was used to measure the health state utilities of the patients. This measure is a preference-based health related quality of life instrument that use multi-attributable utility theory to assign valuations to different health states [9]. Health states are defined by a classification system that include a set of dimensions or attributes of health status, with a number for different levels of each attribute [9]. The version of HUI instrument employed in this study was the interviewer-administered, 4 week recall, HUI3 version. In the HUI3 system, eight attributes including vision, hearing, speech, ambulation, dexterity, emotion, cognition and pain and discomfort define health status. Each attribute has five or six levels, creating 972,000 unique HUI3 health states [10]. Overall scores on the HUI3 range from-0.36 to 1.0, with-0.36 representing the utility of the worst possible HUI3 health state, and 0.0 representing dead and 1.0 representing perfect health. Differences of greater than 0.03 on the HUI2 or HUI3 overall scores may be considered important [10]. In addition to overall scores, single attribute utility scores can be obtained for each attribute of the HUI3, ranging from 0.0 to 1.0, with a score of 0.0 representing the lowest level of functioning [11]. Differences of 0.05 on the single attributes may be considered clinically important [10]. A section to obtain patients' basic demographic and clinical information was attached to the questionnaire. Information of patient's primary activity, whether patient had been off work due to illness or had emergency visit in the last 6 month were obtained through interview. Other variables were obtained from the patient's medical record.

To assess predictors of HUI scores, overall utility score served as the dependent variable while some selected patient's demographic and clinical variables served as independent variables. Study variables found to be correlated to overall utility scores after adjusting for confounding variables were used in a multiple linear regression. Stepwise method was used to model the effect of predictor variables on overall utility scores. Analysis of impact of cardiovascular comordities on overall utility scores of the patients was conducted using ANOVA (LSD for multiple comparisons). Data analysis was conducted with SPSS 11.0^® ^(Chicago, Illinois, USA). A two-tailed significance level of 0.05 was used.

All procedures were carried out according to a study protocol approved by the Local Ethics Committee of University of Nigeria Teaching Hospital Enugu (UNTH/CSA.329.Vol.6) and Ebonyi State University Teaching Hospital (EBSUTH/CMAC/RM/VOL1/046/48). Oral informed consent was obtained. The information about participant's identity was not included with the other data and only the principal investigator had access to this information. No reference to the participant's identity was made at any stage during data analysis

## Results

A total of 635 patients were approached for interview while only 384 participants (60.5%) with either hypertension alone or hypertension-associated complication participated in the study. Lack of time or interest was the major reason given by those that did not participate. Each interview took an average of 6 minutes +/- 1 minute. Majority of the participants were from University of Nigeria Teaching Hospital and the ratio of male to female was almost equal. A small number of the participants had no formal education. Other clinical variables of the participants are shown in Table 1.

**Table 1:** Demographic and Clinical Variables of Participants (n = 384)

Variable	n	Percentage or Median [Interquartile range]
*Hospital*		

University of Nigeria Teaching Hospital	260	67.7

Ebonyi State University Teaching Hospital	124	32.3

*Sex*		

Females	200	52.1

Males	184	47.9

*Educational Status*		

No formal education	8	2.1

Primary	156	40.6

Secondary	132	34.4

Tertiary	88	22.9

*Emergency visit*	120	31.3

*Hospitalization*	228	59.4

*Off work due to illness*	216	56.3

*Number of patient reported symptoms*	384	3.0 [3.0-4.0]

*Age*	384	61.0 [ 54.0-67.8]

*Number of Physician visit last 1 year*	384	6.0 [5.0-9.0]

In Table 2, HUI3 single- and multi-attribute scores for all the hypertensive patients and for those with cardiovascular complications are shown. The overall mean utility score was 0.35+/-0.42. Participants with hypertension alone had the highest mean single- and multi-attribute utility scores. With the exception of the group of hypertensive patients with heart failure, there was a significant difference in the mean multi-attribute utility scores of the other groups compared to patients with hypertension alone. The mean single-attribute utility scores of patient groups with cardiovascular comorbidities differed significantly compared to those with hypertension alone. Apart from the vision attribute, patients with hypertension and CHD had significantly differing mean single attribute scores compared to patients with hypertension alone. Patients with hypertension and stroke did not significantly differ with patients with hypertension alone in the mean hearing and speech attribute scores. Patients with hypertension and heart failure did not differ significantly with patient with hypertension alone except for the mean vision, ambulation and dexterity attributes scores.

**Table 2:** HUI3 single- and multi-attribute scores (n = 384)

Mean ± SD						
	**All participants (n = 384)**	**Hypertension alone (n = 124)**	**Hypertension & HF (n = 104)**	**Hypertension & CHD (n = 52)**	**Hypertension & Stroke (n = 84)**	***p*-value**

Vision	0.86 ± 0.26	0.92 ± 0.16	0.86 ± 0.26*	0.88 ± 0.26	0.77 ± 0.31*	< 0.001

Hearing	0.96 ± 0.14	0.98 ± 0.10	0.95 ± 0.14	0.88 ± 0.22*	0.98 ± 0.11	< 0.001

Speech	0.95 ± 0.14	0.98 ± 0.11	0.95 ± 0.20	0.91 ± 0.11*	0.95 ± 0.10	0.023

Ambulation	0.75 ± 0.37	0.97 ± 0.06	0.86 ± 0.32*	0.55 ± 0.40*	0.46 ± 0.41*	< 0.001

Dexterity	0.83 ± 0.31	1.00 ± 0.00	0.89 ± 0.25*	0.69 ± 0.36*	0.62 ± 0.36*	< 0.001

Emotion	0.74 ± 0.29	0.86 ± 0.18	0.84 ± 0.26	0.66 ± 0.30*	0.59 ± 0.32*	< 0.001

Cognition	0.67 ± 0.29	0.78 ± 0.25	0.76 ± 0.26	0.53 ± 0.24*	0.51 ± 0.25*	< 0.001

Pain	0.56 ± 0.35	0.71 ± 0.26	0.68 ± 0.31	0.34 ± 0.36*	0.34 ± 0.33*	< 0.001

Overall	0.35 ± 0.42	0.57 ± 0.29	0.51 ± 0.38	0.13 ± 0.39*	0.04 ± 0.36*	< 0.001

*HF *heart failure; *CHD *coronary heart disease

*Significantly different (*p *< 0.05) compared to hypertension alone group

In Table 3, patients' variables predicting decrement in overall utility scores are shown. Being a male was associated with statistical significant decrease in overall utility scores. Also, a unit increase in age (years), mean arterial blood pressure (mmHg), emergency visit and loss of work due to illness were associated with statistical significance decrease in overall utility scores. The F-value (106.4, DF = 6) had an associated probability level of *p *< 0.001, showing that the results were unlikely to have arisen by sampling error.

**Table 3:** Predictors of overall utility scores (n = 384)

Variable	β (95% Confidence Interval)	*p*-value
Sex	-0.115 (-0.167--0.030)	0.005*

Age	-0.489 (-0.024--0.016)	< 0.001*

Mean arterial blood pressure	-0.108 (-0.005--0.001)	0.004*

Emergency visit	-0.214 (-0.318--0.121)	< 0.001*

Hospitalization	0.022 (-0.064-0.102)	0.635

Off work due to illness	-0.244 (-0.297--0.134)	< 0.001*

*Significance difference (*p *< 0.05)

## Discussion

Health state utilities of Nigerian hypertensive patients were assessed in this study employing a standard and widely used quality of life measure, the Health Utilities Index Mark 3 [10]. Although a number of generic multi-attribute measures exist for assigning preference weights (utilities) to health states e.g. Quality of Well-Being Index [12], EuroQol EQ-5D [13], the Health Utilities Index is one of the best standardized and most widely respected measures [14]. In our study, the overall utility scores for patients with hypertension alone and those with cormobidites such as stroke, heart failure and coronary heart disease were lower than the overall utility scores that have been reported by other studies. A national health survey conducted in Canada showed a higher overall utility score of 0.82 ± 0.18 for hypertensive participants, 068 ± 0.23 for stroke participants and 0.77 ± 0.21 for participants with heart disease when compared with the results obtained in our survey [15]. Another cross-sectional survey of Australian prisoners using SF-36 data transformed by both the SF-6D and Nichol's method reported utility scores for hypertensive patients of 0.672 ± 0.176 and 0.782 ± 0.155 for SF-6D and Nichol's method of transformation respectively [16]. The lower overall utility score observed in this study could be explained in part by the low socio-economic standards of the population of study. Nigeria is categorized as a developing nation and has lower economic indices compared to developed countries. Besides, the level of health care in Nigerian health facilities is poorer than those of developed countries. Thus it is logical that study participants will perceive their level of health poorly when compared to others living in more affluent environments.

In this study, variables such as being a male as well as increase in age and mean arterial blood pressure, emergency visit to the hospital and loss of work due to illness were all associated with decrease in overall utility scores. Apart from sex (i.e. being a male) all the variables found to lower overall utility scores are expected to cause decrement in health related quality of life. It is important to state that the findings from most quality of life studies show the contrary (i.e. women have lower utilities compared to men) [17,18]. This might need further exploration in order to establish whether the reverse occurs in African countries like Nigeria.

As established in this study, cardiovascular comorbidities were associated with lower utility scores. Specifically, stroke did not have any negative impact on hearing although it did affect vision. This finding is somewhat in agreement with another study which found that stroke and arthritis did not affect speech and cognition [19]. Coronary heart disease affected all the single attributes except vision. This is expected since CHD does not affect vision. Heart failure affected vision, ambulation and dexterity significantly when compared to patients with only hypertension. Although the health related quality of life deficit on vision attribute score is inexplicable, ambulation and dexterity is expected to be affected.

This study had some limitations which should be taken into consideration when interpreting the results. The cross-sectional nature of the study and the convenience sampling may affect the generalizability of the study. Secondly, the study made use of a small sample size as a result language barrier which precluded those that could not understand and speak English language.

## Conclusion

This assessment presents a reference for health state utilities of hypertensive patients with or without associated complications. The utility scores could facilitate health economic evaluations conducted to provide evidence for decision making in health care.

## Competing interests

The authors declare that they have no competing interests.

## Authors' contributions

OIE and CNA designed the study. OIE, EB and CJO actively implemented the study. MOA carried out the statistical analysis. All the authors were involved in articles write-up. All authors read and approved the final manuscript.

## References

[B1] Chobian A Bakris J Black H Cushman W Green L Izzo J Seventh report of the joint National Committee on prevention, detection, evaluation, and treatment of high blood pressure: the JNC 7 report of American Medical Association 2003 11 2890 2560 10.1001/jama.289.19.2560 12748199

[B2] Steyn K Sliwa K Hawken S Commerford P Onen C Damasceno A Risk factors associated with myocardial infarction in Africa: the INTERHEART Africa study Circulation 2005 112 3554 3561 10.1161/CIRCULATIONAHA.105.563452 16330696

[B3] WHO Prospects of research on non-communicable diseases in the African sub-region. World Health Organization 2008 http://www.afro.who.int/dpm/rpc/publicaations/ncdwok.pdf Accessed 12 May

[B4] Khor G Cardiovascular epidemiology in the Asia-Pacific region Asia Pac J Clin Nutr 2001 76 80 10.1111/j.1440-6047.2001.00230.x 11710361

[B5] Vorster H The emergence of cardiovascular disease during urbanisation of Africans Public Health Nutr 2002 239 243 10.1079/phn2001299 12027290

[B6] Addo J Smeeth L Leon DA Hypertension in sub-Saharan Africa: a systematic review Hypertension 2007 50 1012 1018 10.1161/HYPERTENSIONAHA.107.093336 17954720

[B7] Ekwunife O Aguwa C Increasing prevalence of hypertension in Nigeria: A systematic review from 1999-2009 Value Health 2010 13 7 A239 A57321199110

[B8] Saseen JJ Koda-Kimble MA, Young LY, Alldredge BK et al. Essential hypertension Applied Therapeutics: The Clinical Use of Drugs 9 Lipponcott Williams & Wilkins 13p7

[B9] Maddigan SL Feeny DH Johnson JA Construct validity of the RAND-12 and health utilities index mark 2 and 3 in type 2 diabetes Qual Life Res 2004 13 435 488 15085916 10.1023/B:QURE.0000018497.06539.8f

[B10] Feeny DH Furlong WJ Torrance GW Health utilities index: multi-attribute and single-attribute utility functions for the health utilities index mark 3 system Med Care 2002 40 2 113 128 10.1097/00005650-200202000-00006 11802084

[B11] Feeny D Furlong W Boyle M Torrance GW Multi-attribute health status classification systems: health utilities index Pharmacoeconomics 1995 7 490 502 10.2165/00019053-199507060-00004 10155335

[B12] Kaplan R Bush J Berry C Health status: types of validity and the index of well-being Health Serv Res 1976 11 478 507 1030700 PMC1071947

[B13] The EuroQol Group EuroQol-a new facility for the measurement of health-related quality of life Health Policy 1990 16 199 208 10109801 10.1016/0168-8510(90)90421-9

[B14] Torrance G Boyle M Horword S Application of multiattribute utility theory to measure social preferences for health states Operat Res 1982 30 1043 1069 10.1287/opre.30.6.1043 10259643

[B15] Mittmann N Trakas K Risebrough N Liu B Utility scores for chronic conditions in a community-dwelling population Pharmacoeconomics 1999 15 4 369 376 10.2165/00019053-199915040-00004 10537955

[B16] Chong C Li S Nguyen G Sutton A Levy M Butler T Health-state utilities in a prisoner population: a cross-sectional survey Health Qual Life Out 2009 7 78 doi:10.1186/1477-7525-7-78 10.1186/1477-7525-7-78 PMC2741437 19715571

[B17] Bisegger C Cloetta B Bisegger U Abel T Ravens-Sieberer U European Kidscreen group Health-related quality of life: gender differences in childhood and adolescence Social and preventive medicine 50 5 289 291 10.1007/s00038-005-4094-2 16300172

[B18] Riedinger MS Dracup KA Brecht M Padilla G Sarna L SOLVD Investigators Quality of life patients with heart failure: do gender differences exist? heart & lung The Journal of Acute and Critical Care 30 2 105 116 10.1067/mhl.2001.114140 11248713

[B19] Grootendorst P Feeny D William F Health utilities index mark 3: evidence of construct validity for stroke and arthritis in a population health survey Med Care 38 3 290 299 10.1097/00005650-200003000-00006 10718354

